# Supplementary material for: Knowledge, attitudes, and practices among Indonesian urban communities regarding HPV infection, cervical cancer, and HPV vaccination
Source: PLoS One. 2022 May 12;17(5):e0266139. doi: 10.1371/journal.pone.0266139 (PMC9098048; doi:10.1371/journal.pone.0266139)
Supplement: S1 Table — (PDF) [file pone.0266139.s002.pdf]

Knowledge Responses

Table. Knowledge questions and responses from 400 respondents regarding HPV infection, CC, and HPV vaccination

Knowledge Questions	Responds	Men (n=105)		Women (n=295)		Total (n=400)		p-value
		n	%	n	%	N	%	
**Aspect 1: HPV infection and CC**								
K1. CC is caused by a virus infection.	**Yes**	64	61.0	251	85.1	315	**78.8**	**0.000[a]**
	No	41	39.0	44	14.9	85	21.3	
K2. Participants know about the existence of HPV.	**Yes**	52	49.5	201	68.1	253	**63.3**	**0.001[a]**
	No	53	50.5	94	31.9	147	36.8	
K3. HPV infection always causes cancer.	**No**	25	23.8	96	32.5	121	**30.3**	**0.002[a]**
	Yes	22	21.0	95	32.2	117	29.3	
	Don't know	58	55.2	104	35.3	162	40.5	
K4. Men can transmit HPV.	**Yes**	44	41.9	174	54.5	218	**54.5**	**0.002[b]**
	No	4	3.8	10	3.5	14	3.5	
	Don't know	57	54.3	111	42.0	168	42.0	
K5. Men are not at risk for HPV infection.	**False**	34	32.4	135	45.8	169	**42.3**	**0.002[a]**
	True	12	11.4	52	17.6	64	16.0	
	Don't know	59	56.2	108	36.6	167	41.8	
K6. HPV can be transmitted through sexual contact.	**True**	64	61.0	218	73.9	282	**70.5**	**0.008[b]**
	False	2	1.9	11	3.7	13	3.3	
	Don't know	39	37.1	66	22.4	105	26.3	
K7. HPV infection is very rare (sporadic).	**False**	45	42.9	161	54.6	206	**51.5**	**0.003[a]**
	True	5	4.8	33	11.2	38	9.5	
	Don't know	55	52.4	101	34.2	156	39.0	
K8. HPV infection can cause CC.	**True**	62	59.0	240	81.4	302	**75.5**	**0.000[a]**
	Don't know	43	41.0	55	18.6	98	24.5	
K9. Smoking is a risk factor for HPV infection.	**True**	31	29.5	86	29.2	117	**29.3**	0.996[a]
	False	15	14.3	43	14.6	58	14.5	
	Don't know	59	56.2	166	56.3	225	56.3	
**Aspect 2: HPV vaccination**								
K10. Heard about the HPV vaccines.	**Yes**	102	97.1	287	97.3	389	**97.3**	0.585[c]
	No	3	2.9	8	2.7	11	2.8	
K11. CC can be prevented by vaccination.	**Yes**	58	55.2	232	78.6	290	**72.5**	**0.000[a]**
	No	47	44.8	63	21.4	110	27.5	
K12. The HPV vaccine is available in Indonesia.	**Yes**	54	51.4	217	73.6	271	**67.8**	**0.000[a]**
	No	51	48.6	78	26.4	129	32.3	
K13. Know how to access the HPV vaccine.	**Yes**	47	44.8	209	70.8	256	**64.0**	**0.000[a]**
	No	58	55.2	86	29.2	144	36.0	
K14. HPV vaccination has side effects.	**Yes**	20	19.0	63	21.4	83	**20.8**	0.824[a]
	No	16	15.2	48	16.3	64	16.0	
	Don't know	69	65.7	184	62.4	253	63.3	
K15. The risk of infection still exists after HPV vaccination.	**Yes**	41	39.0	150	50.8	191	**47.8**	**0.017[a]**
	No	28	26.7	44	14.9	72	18.0	

	Don't know	36	34.3	101	34.2	137	34.3	
K16. People infected with HPV still need to be vaccinated.	**Yes**	34	32.4	104	35.3	138	**34.5**	0.862[a]
	No	34	32.4	93	31.5	127	31.8	
	Don't know	37	35.2	98	33.2	135	33.8	
K17. HPV-vaccinated people still need regular screening for CC.	**True**	65	61.9	233	79.0	298	**74.5**	**0.000[b]**
	False	0	0.0	6	2.0	6	1.5	
	Don't know	40	38.1	56	19.0	96	24.0	
K18. HPV vaccination can provide 100% protection against CC.	**False**	50	47.6	154	52.2	204	**51.0**	0.659[a]
	True	17	16.2	48	16.3	65	16.3	
	Don't know	38	36.2	93	31.5	131	32.8	
K19. HPV vaccination is only needed by the adults (>30 years).	**False**	48	45.7	181	61.4	229	**57.3**	**0.005[a]**
	True	10	9.5	33	11.2	43	10.8	
	Don't know	47	44.8	81	27.5	128	32.0	
K20. HPV vaccination only takes one dose.	**False**	42	40.0	165	55.9	207	**51.8**	**0.014[a]**
	True	11	10.5	29	9.8	40	10.0	
	Don't know	52	49.5	101	34.2	153	38.3	
K21. After being fully vaccinated against HPV, people can have multiple sex partners.	**False**	60	57.1	215	72.9	275	**68.8**	**0.006[b]**
	True	4	3.8	10	3.4	14	3.5	
	Don't know	41	39.0	70	23.7	111	27.8	

[a]Chi-Square; [b]Mann-Whitney; [c]Fischer's exact test; Percentage of the total column; significant value $p<0.05$
