# Supplementary material for: Knowledge, attitudes, and practices among Indonesian urban communities regarding HPV infection, cervical cancer, and HPV vaccination
Source: PLoS One. 2022 May 12;17(5):e0266139. doi: 10.1371/journal.pone.0266139 (PMC9098048; doi:10.1371/journal.pone.0266139)
Supplement: S2 Table — (PDF) [file pone.0266139.s003.pdf]

# Attitude Responses

**Table.** Attitude questions and responses from 400 respondents regarding HPV infection, CC, and HPV vaccination

| Attitude Questions | Responds | Men (n=105) | | Women (n=295) | | Total (n=400) | | p-value |
|---|---|---|---|---|---|---|---|---|
| | | n | % | n | % | N | % | |
| **Aspect 1: HPV infection and CC** | | | | | | | | |
| A1. HPV is perceived as a dangerous virus. | **Yes** | 88 | 83.8 | 255 | 86.4 | 343 | **85.8** | 0.518[a] |
| | No | 3 | 2.9 | 6 | 2.0 | 9 | 2.3 | |
| | Don't know | 14 | 13.3 | 34 | 11.5 | 48 | 12.0 | |
| A2. Participants considered their susceptibility to HPV infection. | **Yes** | 12 | 11.4 | 44 | 14.9 | 56 | **14.0** | 0.282[b] |
| | Maybe | 17 | 16.2 | 51 | 17.3 | 68 | **17.0** | |
| | Unsure | 28 | 26.7 | 96 | 32.5 | 124 | 31.0 | |
| | No | 48 | 45.7 | 104 | 35.3 | 152 | 38.0 | |
| A3. Health is considered an essential aspect of life. | **Absolutely agree** | 90 | 85.7 | 268 | 90.8 | 358 | **89.5** | 0.141[b] |
| | Agree | 15 | 14.3 | 27 | 9.2 | 42 | 10.5 | |
| A4. Prevention is the primary key against disease. | **Absolutely agree** | 90 | 85.7 | 262 | 88.8 | 352 | **88.0** | 0.409[a] |
| | Agree | 15 | 14.3 | 32 | 10.8 | 47 | **11.8** | |
| | Disagree | 0 | 0.0 | 1 | 0.3 | 1 | 0.3 | |
| A5. Worried about partner or closed family will get CC | **Absolutely agree** | 43 | 41.0 | 155 | 52.5 | 198 | **49.5** | **0.027[a]** |
| | Agree | 48 | 45.7 | 116 | 39.3 | 164 | **41.0** | |
| | Disagree | 12 | 11.4 | 19 | 6.4 | 31 | 7.8 | |
| | Absolutely disagree | 2 | 1.9 | 5 | 1.7 | 7 | 1.8 | |
| **Aspect 2: HPV vaccination** | | | | | | | | |
| A6. Support for use of HPV vaccines for children | **Yes** | 103 | 98.1 | 288 | 97.6 | 391 | **97.8** | 0.565[c] |
| | No | 2 | 1.9 | 7 | 2.4 | 9 | 2.3 | |
| A7. Support for use of HPV vaccines for adults | **Yes** | 101 | 96.2 | 292 | 99.0 | 393 | **98.2** | 0.081[c] |
| | No | 4 | 3.8 | 3 | 1.0 | 7 | 1.8 | |
| A8. Willingness to get HPV vaccinated | **Yes** | 43 | 41.0 | 225 | 76.3 | 268 | **67.0** | **0.000[b]** |
| | Maybe | 21 | 20.0 | 27 | 9.2 | 48 | **12.0** | |
| | Unsure | 22 | 21.0 | 31 | 10.5 | 53 | 13.3 | |
| | No | 19 | 18.1 | 12 | 4.1 | 31 | 7.8 | |
| A9. My partner or closed family must be vaccinated against HPV | **Yes** | 75 | 71.4 | 223 | 75.6 | 298 | **74.5** | 0.562[b] |
| | Maybe | 16 | 15.2 | 30 | 10.2 | 46 | **11.5** | |
| | Unsure | 8 | 7.6 | 22 | 7.5 | 30 | 7.5 | |
| | No | 6 | 5.7 | 20 | 6.8 | 26 | 6.5 | |
| A10. Participants fear injections in general | **Absolutely disagree** | 31 | 29.5 | 75 | 25.4 | 106 | **26.5** | 0.614[b] |
| | Disagree | 44 | 41.9 | 133 | 45.1 | 177 | **44.3** | |
| | Agree | 19 | 18.1 | 64 | 21.7 | 83 | 20.8 | |
| | Absolutely agree | 11 | 10.5 | 23 | 7.8 | 34 | 8.5 | |
| A11. Worriness about the side effects of the HPV vaccine | **Absolutely disagree** | 9 | 8.6 | 20 | 6.8 | 29 | **7.3** | 0.617[b] |
| | Disagree | 47 | 44.8 | 117 | 39.7 | 164 | **41.0** | |
| | Agree | 43 | 41.0 | 134 | 45.4 | 177 | 44.3 | |
| | Absolutely agree | 6 | 5.7 | 24 | 8.1 | 30 | 7.5 | |
| A12. Participants is not fearing to get vaccinated with HPV. | **Absolutely agree** | 32 | 30.5 | 102 | 34.6 | 134 | **33.5** | 0.346[a] |

| | | | | | | | |
|---|---|---|---|---|---|---|---|
| | Agree | 56 | 53.3 | 155 | 52.5 | 211 | **52.8** |
| | Disagree | 16 | 15.2 | 33 | 11.2 | 49 | 12.3 |
| | Absolutely disagree | 1 | 1.0 | 5 | 1.7 | 6 | 1.5 |
