# Supplementary material for: Knowledge, attitudes, and practices among Indonesian urban communities regarding HPV infection, cervical cancer, and HPV vaccination
Source: PLoS One. 2022 May 12;17(5):e0266139. doi: 10.1371/journal.pone.0266139 (PMC9098048; doi:10.1371/journal.pone.0266139)
Supplement: S3 Table — (PDF) [file pone.0266139.s004.pdf]

Practice Responses

Table. Practice questions and responses from 400 respondents regarding HPV infection, CC, and HPV vaccination

Practice Questions	Responds	Men (n=105)		Women (n=295)		Total (n=400)		p-value
		n	%	n	%	N	%	
P1. My parents make decisions on my HPV vaccinations	**Absolutely disagree**	8	7.6	26	8.8	34.2	**8.5**	0.912[a]
	Disagree	43	41.0	122	41.4	165	**41.3**	
	Agree	37	35.2	107	36.3	144	36.0	
	Absolutely agree	17	16.2	40	13.6	57	14.2	
P2. My friends influence my decision to vaccinate against HPV	**Absolutely disagree**	21	20.0	56	19.0	77	**19.3**	0.459[b]
	Disagree	58	55.2	185	62.7	243	**60.8**	
	Agree	24	22.9	48	16.3	72	18.0	
	Absolutely agree	2	1.9	6	2.0	8	2.0	
P3. HPV vaccine does not conflict with my religion	**Absolutely agree**	37	35.2	128	43.4	165	**41.3**	**0.035[b]**
	Agree	45	42.9	132	44.7	177	**44.3**	
	Disagree	20	19.0	28	9.5	48	12.0	
	Absolutely disagree	3	2.9	7	2.4	10	2.5	
P4. I have been vaccinated against HPV at least once	**Yes**	6	5.7	63	21.4	69	**17.3**	**0.000[a]**
	No	99	94.3	232	78.6	331	82.8	
P5. I am intend and get ready to get the HPV vaccine.	**Absolutely agree**	20	19.0	120	40.7	140	**35.0**	**0.000[b]**
	Agree	53	50.5	147	49.8	200	**50.0**	
	Disagree	31	29.5	28	9.5	59	14.8	
	Absolutely disagree	1	1.0	0	0.0	1	0.3	
P6. I will tell my family and partner about CC and HPV vaccine	**Absolutely agree**	24	22.9	126	42.7	150	**37.5**	**0.000[b]**
	Agree	68	64.8	157	53.2	225	**56.3**	
	Disagree	12	11.4	11	3.7	23	5.8	
	Absolutely disagree	1	1.0	1	0.3	2	0.5	
P7. I want to get more information about HPV, CC, and vaccination.	**Absolutely agree**	34	32.4	143	48.5	177	**44.3**	**0.000[b]**
	Agree	59	56.2	146	49.5	205	**51.3**	
	Disagree	9	8.6	4	1.4	13	3.3	
	Absolutely disagree	3	2.9	2	0.7	5	1.3	

[a]Chi-Square; [b]Mann-Whitney; Percentage of the total column; significant value $p<0.05$
