# Supplementary material for: Knowledge, attitudes, and practices among Indonesian urban communities regarding HPV infection, cervical cancer, and HPV vaccination
Source: PLoS One. 2022 May 12;17(5):e0266139. doi: 10.1371/journal.pone.0266139 (PMC9098048; doi:10.1371/journal.pone.0266139)
Supplement: S1 File — (PDF) [file pone.0266139.s005.pdf]

Questionnaire and Scoring Scheme

Knowledge

Items	Questions	Answers	Score	Dichotomization of Answers
Aspect 1	HPV infection and cervical cancer			
K1	Do you know that cervical cancer is a disease caused by a viral infection?	**Yes**	2	Good
		No	0	Poor
K2	Do you know about the existence of the Human Papilloma Virus (HPV)?	**Yes**	2	Good
		No	0	Poor
K3	Does HPV infection always cause cancer?	**No**	2	Good
		Yes	0	Poor
		Don't Know	0	
K4	Men can transmit HPV.	**Yes**	2	Good
		No	0	Poor
		Don't Know	0	
K5	Men are not at risk for HPV infection.	**False**	2	Good
		True	0	Poor
		Don't Know	0	
K6	HPV can be transmitted through sexual contact.	**True**	2	Good
		False	0	Poor
		Don't know	0	
K7	HPV infection is very rare (sporadic).	**False**	2	Good
		True	0	Poor
		Don't know	0	
K8	HPV infection can cause cervical cancer.	**True**	2	Good
		False	0	Poor
		Don't know	0	
K9	Smoking is a risk factor for HPV infection.	**True**	2	Good
		False	0	Poor
		Don't know	0	
	Total Knowledge Score for Aspect 1		18/18 x 100%	
Aspect 2	HPV Vaccination			
K10	Have you ever heard about the HPV vaccine?	**Yes**	2	Good
		No	0	Poor
K11	Have you ever heard that vaccination can prevent cervical cancer?	**Yes**	2	Good
		No	0	Poor
K12	Do you know that the cervical cancer vaccine or HPV vaccine is already available in Indonesia?	**Yes**	2	Good
		No	0	Poor
K13	Do you know whom you should reach or contact if you want to get cervical cancer (HPV) vaccination?	**Yes**	2	Good
		No	0	Poor
K14	Do you think cervical cancer (HPV) vaccine has side effects?	**Yes**	2	Good
		No	0	Poor
		Don't know	0	
K15	Do you think the HPV virus can still infect someone if they have been vaccinated?	**Yes**	2	Good
		No	0	Poor
		Don't know	0	
K16	Do you think that someone who has been infected with HPV still needs to be vaccinated?	**Yes**	2	Good
		No	0	Poor
		Don't know	0	
K17		**True**	2	Good

	Women who have been vaccinated still need to have periodic early detection (screening) examinations (Pap's Smear / IVA) later.	False	0	Poor
		Don’t know	0	
K18	Vaccination can provide 100% protection against cervical cancer.	**False**	2	Good
		True	0	Poor
		Don’t know	0	
K19	HPV vaccination is only needed by adults (>30 years).	**False**	2	Good
		True	0	Poor
		Don’t know	0	
K20	Only with one dose of vaccination, I will get complete protection against cervical cancer.	**False**	2	Good
		True	0	Poor
		Don’t know	0	
K21	After receiving the total dose of the HPV vaccine, sexual intercourse with multiple partners is allowed.	**False**	2	Good
		True	0	Poor
		Don’t know	0	
	Total Knowledge Score for Aspect 2		24/24 x 100%	
	Total Knowledge Score Aspect 1 + 2		42/42 x 100%	
	Score ≥60% = Good; <60% = Poor			

Notes: The words in bold in the answer column indicate the appropriate answer.

Attitudes

Items	Questions	Answers	Score	Dichotomization of Answers
Aspect 1	HPV infection and cervical cancer			
A1	Do you think that HPV is a dangerous virus?	**Yes**	2	Positive
		No	0	Negative
		Don’t know	0	
A2	In your opinion, are you susceptible to HPV infection?	**Yes**	2	Positive
		Maybe	1	
		Unsure	0	Negative
		No	0	
A3	I believe that health is one of the most important aspects of my life.	**Absolutely agree**	2	Positive
		Agree	1	
		Disagree	0	Negative
		Absolutely disagree	0	
A4	Prevention is the primary key in fighting disease.	**Absolutely agree**	2	Positive
		Agree	1	
		Disagree	0	Negative
		Absolutely disagree	0	
A5	I am worried that I/my partner/people close to me may develop cervical cancer in the future.	**Absolutely agree**	2	Positive
		Agree	1	
		Disagree	0	Negative
		Absolutely disagree	0	
	Total Attitude Score for Aspect 1		10/10 x 100%	
Aspect 2	HPV Vaccination			
A6	Do you support HPV vaccination for children?	**Yes**	2	Positive
		No	0	Negative
A7	Do you support HPV vaccination for adults?	**Yes**	2	Positive
		No	0	Negative
A8	Do you want to get the HPV vaccination?	**Yes**	2	Positive
		Maybe	1	

		Unsure	0	Negative
		No	0	
A9	Do you think that you should also get HPV vaccinated in the future?	**Yes**	2	Positive
		Maybe	1	
		Unsure	0	Negative
		No	0	
A10	I am afraid of injections.	**Absolutely disagree**	2	Positive
		Disagree	1	
		Agree	0	Negative
		Absolutely agree	0	
A11	I am worried about the side effects of the HPV vaccine.	**Absolutely disagree**	2	Positive
		Disagree	1	
		Agree	0	Negative
		Absolutely agree	0	
A12	I am not afraid if I get HPV vaccinated.	**Absolutely agree**	2	Positive
		Agree	1	
		Disagree	0	Negative
		Absolutely disagree	0	
	**Total Attitude Score for Aspect 2**		**14/14 x 100%**	
	**Total Attitude Score Aspect 1 + 2**		**24/24 x 100%**	
	**Score ≥60% = Positive; <60% = Negative**			

Notes: The words in bold in the answer column indicate the appropriate answer.

Practices

Items	Questions	Answers	Score	Dichotomization of Answers
P1	My parents make my vaccination decisions (including HPV vaccination).	**Absolutely disagree**	2	Favorable
		Disagree	1	
		Agree	0	Unfavorable
		Absolutely agree	0	
P2	My friends influenced my decision to receive the HPV vaccination.	**Absolutely disagree**	2	Favorable
		Disagree	1	
		Agree	0	Unfavorable
		Absolutely agree	0	
P3	HPV vaccine does not conflict with my religion.	**Absolutely agree**	2	Favorable
		Agree	1	
		Disagree	0	Unfavorable
		Absolutely disagree	0	
P4	I have been vaccinated against HPV at least one dose.	**Yes**	2	Favorable
		No	0	Unfavorable
P5	I am willing to get the HPV vaccination.	**Absolutely agree**	2	Favorable
		Agree	1	
		Disagree	0	Unfavorable
		Absolutely disagree	0	
P6	I am willing to tell my family and partner about the risks of cervical cancer and information about the HPV vaccine.	**Absolutely agree**	2	Favorable
		Agree	1	
		Disagree	0	Unfavorable
		Absolutely disagree	0	
P7	I want to get more information about HPV.	**Absolutely agree**	2	Favorable
		Agree	1	
		Disagree	0	Unfavorable
		Absolutely disagree	0	

	Total Practice Score	14/14 x 100%	
	Score ≥60% = Favorable; <60% = Unfavorable		

Notes: The words in bold in the answer column indicate the appropriate answer.
