# Supplementary material for: Knowledge, attitudes, and practices among Indonesian urban communities regarding HPV infection, cervical cancer, and HPV vaccination
Source: PLoS One. 2022 May 12;17(5):e0266139. doi: 10.1371/journal.pone.0266139 (PMC9098048; doi:10.1371/journal.pone.0266139)
Supplement: Attachment — Submitted filename: Review to the Article PLOSONE_ KAP and cervical cancer.pdf [file pone.0266139.s007.pdf]

Review to the Article

Knowledge, attitudes, and practices among Indonesian urban communities regarding HPV infection, cervical cancer, and HPV vaccination (PONE-D-21-35609)

Mugi Wahidin

1. Title
   Title should in line with the study population which is Jakarta citizens, not all urban communities in Indonesia. We suggest you to revise it.
2. Abstract
   a. Background: Revise the word "no study explored…". There are many studies about HPV and cervical cancer. It's better if you write there is limitation of study about it, but you should provide the studies in the background
   b. Aim: what is the aim of the study? Association of KAP towards HPV infection, CC, and HPV vaccine? It's different with the result that is association of socio-demographic with KAP in terms of HPV infection, CC, and HPV vaccination. Make it clear.
   c. Method: add who the respondent are (Jakarta), sampling technique, how to get the respondent and sample size, how to collect data and when you did
   d. Result: Make it simple as aim of the study, not all result. We suggest to delete median, just straight to say high or poor (KAP)
   e. Conclusion: make it more specific related to the aim of the study.
3. Background
   Explain why you choose urban communities. Cervical cancer is a disease not only in urban, but also in rural communities. Provide evidence, such as comparison incidence or prevalence in urban and rural areas, and related studies of KAP and cervical cancer. Again, word " no study explored…" should be revised
4. Method
   a. Describe how to spread the link/web in social media to invite the respondents.
   b. Explain how sample size is counted, there are several independent and dependent variables to be considered.
   c. Add information of time of data collection (is it more than a year from March 2020 – August 2021?). Is there any inclusion criteria/restriction of age? How a teenager of age of 11 years become respondent?
   d. In assessment tools, we suggest to move contributory from authors to specific section, not in method.
   e. In statistical analysis, add information of normality test, due to use of Spearman Correlation test. Add steps of multivariate logistic regression analysis, how the modeling constructed and how to get the final model (do you put the final model in the result?)
5. Result
   a. Table 2 – 4 is not needed, its only step to get table 4. We suggest deleting table 2-4.

    b. Table 6-9: are they the final model? You should give explanation. Put only the significant variables in the final model.
6. Discussion
    a. The age of respondents majority in age of 11-30 (80%), the must a specific recommendation in this age
    b. Add limitation of the study in terms of sampling technique (snowball), that can not fully represent the population of Jakarta citizens/urban communities. The limitation of characteristic especially age, so result of the study may not be generalized to all ages.
7. Conclusion
   Elaborate the most interesting findings related to the aim of the study. We suggest not to use term "domain" that is not easy to understand.
