# Supplementary material for: Knowledge, attitudes, and practices among Indonesian urban communities regarding HPV infection, cervical cancer, and HPV vaccination
Source: PLoS One. 2022 May 12;17(5):e0266139. doi: 10.1371/journal.pone.0266139 (PMC9098048; doi:10.1371/journal.pone.0266139)
Supplement: Attachment — Submitted filename: Regarding Winarto et al.docx [file pone.0266139.s006.docx]

Winarto et al “Knowledge, attitudes, and practices among Indonesian urban communities regarding HPV infection, cervical cancer, and HPV vaccination”

Thankyou for asking me to review this manuscript.

Because of the high prevalence of cervical cancer in Indonesia, this is an important topic for both Indonesian women and men.

The concepts included in the study are excellent.

Of special interest to Western readers is the demonstration by this Indonesian group of the use of almost cost free social media to conduct an important study.

As shown in this study, HPV vaccination rates are low for both women and men (particularly men).

The results of the study indicate that an education campaign is required to increase the HPV vaccination rates.

Congratulations to the authors for their interest in this important topic and for their ability to conduct a study without cost. Well done!

In my view, after revision, the study should be published.

Limitations and suggestions

The following comments and suggestions are intended to be helpful. They are not criticisms. They mainly concern ways in which to improve the presentation of the manuscript.

1. The manuscript needs a review to improve the English writing.

2. There is an excessive use of acronyms (particularly in the abstract). These are distracting for the reader. There is advantage in using repeated full spelling of unusual phrases such as “… knowledge, attitudes, and practices (KAP)…”.

3. The methods used to select the participants in the surveys is not sound from a statistical basis. The participants are far from representative of the Indonesian urban population. For this study this is not important but the limitations need to be recognised and included in the methods.

4. Costs of HPV vaccines. I am not well informed but I understand that one reason for the low HPV vaccination rates in Indonesia is the cost to the recipient and their families. Income levels is low at $350 US dollars per month. Western readers are accustomed to much higher incomes This payment should be presented as per month each time it is included in the manuscript.

5. The manuscript is much too detailed. It is hard work reading the detail. I suggest much of the detailed data could be in Supplementary Tables. In addition much detailed data could be excluded from the narrative.

6. I suggest that the authors include a comparison of the Indonesian guidelines for HPV vaccination with those of the United States Center for Disease Control. In the US HPV vaccination is recommended for young teenagers. The US guide also recommendation no HPV vaccination for people over the age of 26 (because of the likelihood of prior HPV infections). The situation may be very different in Indonesia. Sexual behaviour (the most common means of HPV transmission) is presumably different.

This reviewer is not from the US but many readers of this Journal are American.

7. I suggest the authors include the risk of HPVs in male prostate cancer in their discussions section. This may alter the interest in HPV vaccination by male Indonesians. (see recent reviews in this Journal). Also women catch HPV infections from men. Based on this study, the HPV vaccinations are much lower in males than in females
