# Supplementary material for: Knowledge, attitudes, and practices among Indonesian urban communities regarding HPV infection, cervical cancer, and HPV vaccination
Source: PLoS One. 2022 May 12;17(5):e0266139. doi: 10.1371/journal.pone.0266139 (PMC9098048; doi:10.1371/journal.pone.0266139)
Supplement: Attachment — Submitted filename: Response to Reviewers.docx [file pone.0266139.s008.docx]

**February 24, 2022**

**Response to Reviewers**

**Dear**

Ivan Sabol, PhD

Academic Editor of PLOS ONE

The authors truly respect the time and effort you dedicated to providing constructive and insightful feedback on our manuscript. We appreciate the valuable input which allows us to revise our manuscript titled **"Knowledge, Attitudes, and Practices Among Indonesian Urban Communities Regarding HPV Infection, Cervical cancer, and HPV Vaccination**" (Manuscript ID = PONE-D-21-35609) for publication in PLOS ONE.

We have made changes within the manuscript, incorporating suggestions from reviewers. Regarding your notes, we tried to address the study shortcomings regarding the survey methodology raised by all reviewers, especially about the inclusion/exclusion criteria, implementation/response collection, and representation. We also attempted to fix the language issues and shorten the text or move parts into supplemental material to make it more concise. Details of the changes we made can be seen through the Track Changes feature. We outline our responses in answering the reviewer's comments along with the detailed explanations in the sequence table below. We believe that all the comments from reviewers and editors help us improve our manuscript and are more valuable to the reader.

**#Editor**

**Table 1.** Summary of revision based on Comments from Editor

| **No.** | **Suggestions** | **Response from the Author** |
| --- | --- | --- |
| 1. | Please aim to address the study shortcomings regarding the survey methodology raised by all reviewers, especially about the inclusion/exclusion criteria, implementation/ response collection, and representativeness. | Thank you for your emphasize points for our revisions. Following editor and reviewer comments, we have addressed several issues on our manuscript, including:   1. Fixing the survey methodology 2. Adding inclusion and exclusion criteria 3. Making more explicit explanations about the response collection technique 4. Describing issues about limitations regarding representation according to the methods used in this study: online survey analysis with snowball sampling technique   **Current position of revision in new version of Manuscript (without track changes mode): Line 188-264, p7-10 and Line 643-671, p27-28** |
| 2. | Try to address the language issues and, if possible, shorten the text or move parts to the supplemental material to make it more concise.  The manuscript would benefit from some shortening as well as some language revisions | Thank you for your detailed feedback and constructive suggestion. We have tried our best to shorten the manuscript. However, due to several additional reviewers' requests, we also have added new information to the text body. We also have tried to be as effective with our language as possible.  Additionally, we have proofread our manuscript again regarding your comments on the need for language revisions. We are aware that we are not native speakers of English, and thus we used grammar help software such as Grammarly and help from experts in Academic English writing to revise our language. Hopefully, it has met your expectation |
| 3. | We note that you have stated that you will provide repository information for your data at acceptance. Should your manuscript be accepted for publication, we will hold it until you provide the relevant accession numbers or DOIs necessary to access your data. If you wish to make changes to your Data Availability statement, please describe these changes in your cover letter, and we will update your Data Availability statement to reflect the information you provide. | Thank you for noticing this matter. We have uploaded our dataset using DRYADS, and the current status for the submission is still "in progress," with the DOI number: https://doi.org/10.5061/dryad.ns1rn8pv4. However, we have not submitted this dataset because we will wait until this manuscript has been accepted on PLOS One. Please allow us to proceed with the final dataset at the acceptance of our paper.  Here is our submission process for the dataset on DRYAD:     |
| 4. | Please include captions for your Supporting Information files at the end of your manuscript, and update any in-text citations to match accordingly. Please see our Supporting Information guidelines for more information: http://journals.plos.org/plosone/s/supporting-information. | Thank you for pointing this out. We have followed your guidelines, and please allow us to attach the revised versions of our supplementary files. We modified previous supplementary files and added the number of files due to the reviewer comments to make the main text body briefer. |
| 5. | Reference 20 at pate 7 L171 appears to be irrelevant for the study performed. However, additional information on the study design might be helpful, i., i.e., how potential bias was avoided or corrected for. While not an expert on survey design, I am aware of several biases in online questionnaires myself and would inquire whether particular steps were taken to minimize the bias  Please see the following links for some of the problems https://www.d8aspring.com/blog/4-types-of-biases-in-online-surveys-and-how-to-address-them  The Importance of Selection Bias in Internet Surveys. https://www.scirp.org/journal/paperinformation.aspx?paperid=67313  Participation Bias Assessment in Three High-Impact Journals. https://journals.sagepub.com/doi/full/10.1177/2158244013511260  The authors only state P26 L563 "Similar to many survey-based analyses; our research is prone to a response bias" which seems inadequate considering the length of the manuscript | Thank you for asking about this matter. We cited reference 20 (Martinez R, Masron IN. Jakarta: A city of cities. Cities. 2020;106:102868) to explain the domicile and residency criteria for an urban citizen in Jakarta. Since strictly speaking, the definition of a resident of Jakarta is someone who has lived in Jakarta for six months. This cited reference also explains Jakarta's situation and status in Indonesia as one of Indonesia's most significant metropolitan cities, which has a population quite representative of other Indonesian cities and a routine destination for transmigration from other cities.  Thank you for the comment that we need to emphasize discussions on avoiding biases inherent to the study design. We have therefore included several new points, such as:   1. Stating the inclusion and exclusion criteria for respondents in the paper, including the requirements that participants should have a basic level of literacy and internet access 2. The inclusion criteria for Jakarta residency would only include people who have spent a minimum of six months as residents of Jakarta. 3. We also added that further verification was done to ensure our samples were indeed based in Jakarta. After filling out the questionnaire, the subjects were approached through text message or phone calls to ensure that they were eligible to our inclusion criteria, mainly to ensure that they were Jakarta citizens. 4. We have stated further details on our conduction of an exponential non-discriminative snowball sampling, which is a study design allowing for broad sample characteristics [1]. 5. To minimize the effect of Snowball sampling, which is not a random sampling technique and avoids an unrepresentative sample, we have tried to distribute the questionnaire randomly through social media, with no researcher intention of choosing the respondents subjectively [2]. 6. We have tried to anticipate respondents having no response to our inquiry by making the questions unskippable in the online forms system (Google Forms) 7. We have also added a caveat that despite the seeming representation issue regarding our sampling technique, our sample pool remains valuable, representative, and significant in representing the urban citizens of Indonesia since Jakarta is the largest metropolitan city in Indonesia which attracts significant amounts of its population through transmigration from other Indonesian cities.   **Current position of revision in new version of Manuscript (without track changes mode): Line 188-264, p7-10** |
| 7. | While it is interesting to note what factors were associated with positive attitudes, it might be interesting to look at the problem other way around. The authors note that P25 L532 "It is essential to identify influenced factors regarding vaccinations uptakes as a basis of policy to improve vaccination rates." Thus it might be important to find factors most associated with vaccine hesitancy (or lack of knowledge or poor attitudes) and focus policy towards those groups? The current study well describes people not needing policy attention? | Thank you for pointing this out. At this point, we have listed the several factors that influence positive attitudes **(Table 4):**   1. Mother's educational background (OR=4.13, p<0.001) 2. Younger age <25 years old (OR=1.86, p<0.05) 3. Prior good knowledge (OR=2.96, p<0.001).     Furthermore, we also discovered factors that more specifically influenced the vaccinations uptakes **(Table 5):**   1. Female sex (OR=3.88, p<0.05) 2. Mother's educational background (OR=2.42, p<0.01) 3. Working with high wages (OR=2.78, p<0.01) 4. Prior good knowledge (OR=2.81, p<0.01).   Meanwhile, factors that influence the intention/ readiness to get HPV vaccine **(Table 5)** are:   1. Female sex (OR=3.48, p<0.01) 2. Mother's educational background (OR=2.42, p<0.01) 3. Had excellent knowledge (OR=2.77, p<0.01) 4. Having a positive attitude in which the respondents are convinced about the efficacy of the HPV vaccine (OR=8.04, p<0.001)   Positive attitude factors which influence vaccine uptake **(Table 6)** are:   1. Having the willingness to get the vaccine (OR=4.73, p<0.001) 2. Not worrying about side effects (OR=4.63, p<0.001)   Moreover, positive attitude factors which affect intention/readiness to get the HPV vaccine **(Table 6)** are:   1. Perceived the dangers of HPV (OR=2.89, p<0.05) 2. Willingness to get vaccinated from internal motivation (OR=6.61, p<0.001) 3. No fear of HPV vaccination (OR=3.65, p<0.01)   In our perspective, the conceptualization of this study follows the hypothesis: K --> A --> P concept, where good knowledge will support attitude, and a positive attitude will support favorable practice. To enhance knowledge and attitude from the patient's perspective, they could use their digital device to increase their knowledge and awareness, especially among the young generation, about HPV-related disease and CC since age is also a significant predictor. Government and healthcare professionals can support this step by providing a digital health education platform.  From the patient side, we also believe that as family members, mothers could enhance their capacity to support their children's health status, including HPV and CC issues. Moreover, the medical profession and government should empower mothers' KAP about this issue to encourage their children to take vaccines since this factor was significant in several analyses (including our analyses).  Another significant issue is regarding the expensive cost of vaccines. The government should have a special notice on this issue and provide subsidies, information support, etc. This vaccine for now only targets school children in several Indonesian cities for free.  Therefore, these lines of discussion have been added to the main manuscript, and we hope we could further emphasize the various aspects we could avoid vaccine hesitancy and lack of awareness in our population.  **Current position of revision in new version of Manuscript (without track changes mode): Line 689, p28** |
| 8. | While revising your submission, please upload your figure files to the Preflight Analysis and Conversion Engine (PACE) digital diagnostic tool, https://pacev2.apexcovantage.com/. PACE helps ensure that figures meet PLOS requirements. To use PACE, you must first register as a user. Registration is free. Then, log in and navigate to the UPLOAD tab, where you will find detailed instructions on how to use the tool. If you encounter any issues or have any questions when using PACE, please email PLOS at figures@plos.org. Please note that Supporting Information files do not need this step. | Thank you. We have used PACE to adjust our figures and ensure our figures are valid following your requirements. We have attached the proper revised figures after processing in PACE.  Here is the screenshot while using PACE to convert our figure following PLOS One guidelines. Also, we have downloaded the preview figure for PLOS One published paper from PACE.   |

**#Reviewer Number 1**

**Comments:** Because of the high prevalence of cervical cancer in Indonesia, this is an important topic for both Indonesian women and men. The concepts included in the study are excellent. Of special interest to Western readers is the demonstration by this Indonesian group of the use of almost cost free social media to conduct an important study. As shown in this study, HPV vaccination rates are low for both women and men (particularly men). The results of the study indicate that an education campaign is required to increase the HPV vaccination rates. Congratulations to the authors for their interest in this important topic and for their ability to conduct a study without cost. Well done! In my view, after revision, the study should be published.

**Author's Responses:** Thank you for your generous comment and suggestions to improve our paper. We appreciate the details you shared about areas we can improve upon—this insight will help us significantly improve our paper. Hopefully, this article could enlighten readers about the HPV vaccination atmosphere in Indonesia among men and women. We admire your valuable input that this paper will be valuable in both low-to-middle income and developed countries. Living in a developing country is struggling; thus, all the research was conducted efficiently and effectively to minimize cost. Although it has several limitations arising from several aspects, we hope it still serves the best idea and research interests for readers worldwide involved in this topic. All your suggestions will be discussed in the sequence below.

**Table 2.** Summary of revision based on Comments from Reviewer 1

| **No.** | **Suggestions** | **Response from the Author** |
| --- | --- | --- |
| 1. | The manuscript needs a review to improve the English writing | Thank you for your recommendation. We have proofread and checked the entirety of our papers to correct grammar mistakes, particularly in the Results section. As non-native speakers, we have opted to check our paper using applications such as Grammarly and support from experts in academic English, particularly in writing scientific papers. Hopefully, it has sufficiently met your expectations. |
| 2. | There is an excessive use of acronyms (particularly in the abstract). These are distracting for the reader. There is advantage in using repeated full spelling of unusual phrases such as "… knowledge, attitudes, and practices (KAP)…". | Thank you for your detailed feedback. All acronyms in our abstract were proposed to minimize the word count. We cannot present many results in one short abstract without shortening words to compensate for the readability criteria. However, we have tried to correct our abstract as per your suggestion to reduce the unusual phrases and acronyms.  **Current position of revision in new version of Manuscript (without track changes mode): Line 109-133, p5** |
| 3. | a. The methods used to select the participants in the surveys is not sound from a statistical basis.  b. The participants are far from representative of the Indonesian urban population. For this study this is not important but the limitations need to be recognised and included in the methods. | We appreciate the time you have committed to providing detailed and constructive feedback. Here we provide some revisions regarding the methods you ask:   a **Calculation of sample**   We used the sampling calculation based on a statistical basis using a formula of the differences in two proportions in observational studies for descriptive and categorical data [3–6]. However, this paper did not detail the formula due to word number limitations following PLOS One guidelines. The detail of the formula for our calculation sample in this research was:  $n= \frac{{Z\alpha}^{2} \times P \times Q}{d^{2}}$   - n = the size of the sample used - α = generalization of wrong, assumed to be 0.05 - Zα = 1.96 (for α-value= 0.05). This is a standard value for alpha, and the value was obtained from the Z-table normal curve - P = the proportion of categories or conditions or health states that is the point of interest (sought state). The value should be obtained from the literature, preliminary studies, or assumption. However, there is no previous research that was relevant to our research; the assumption of a proportion of 0.5 was used - Q = 1-P, thus the value was 0.5 - d = the desired absolute level of accuracy or the precision of the estimate, namely the precision of the study as an error that is still acceptable (set by the researcher based on logical and enforceable principles), thus we decided the value was 0.05   $n= \frac{{1.96}^{2} \times0.5 \times0.5}{{0.05}^{2}}=196$  Nevertheless, 400 respondents participated in this study.  **Current position of revision in new version of Manuscript (without track changes mode): Line 194-199, p8**   b **Limitation of the survey sample**   We admit that using the online survey and snowball sampling method raises several limitations, which have been addressed in our paper. The limitations regarding our method have been put in the last paragraph before the conclusion section. We are aware that an online survey was welcome with several limitations, including only being completed by the portion of the population literate and competent enough to interpret the questions by themselves. There is also potential for unanswered questions, although this is anticipated by making the questions unskippable in the online forms system. Unfortunately, we could not anticipate the under-representability of unprivileged members of the society who might have experienced difficulties in accessing the Internet, especially in the COVID-19 pandemic times.  Regardless of its benefits, snowball sampling is admittedly minimal. It is a nonrandom sampling method, which might lessen the representation of the entire population and preclude generalizations to the entire city population. The snowball technique also has little control over the sampling method and removes the researcher from the center of the sampling process, which can mean that the survey travels further but might also cause sampling bias because respondents tend to forward the survey to peers with similar traits and characteristics. However, we anticipated this obstacle by conducting exponential non-discriminative snowball sampling, which allows us to have a broad characteristics sample [1]. We distribute the questionnaire randomly through social media, and researchers have no intention to choose the respondents subjectively [2].  Despite those limitations, our ﬁndings are consistent with the outcomes of several studies with similar study methods [7,8]. Due to the scarce data in this field, our work can contribute to the evidence of an association between knowledge, attitude, and practice regarding HPV infection, CC, and HPV vaccination. Our sample from Indonesia urban citizens pictured various background socioeconomic strata and an analysis of their influences on a proper understanding of CC and HPV infection prevention-related issues.  **Current position of revision in new version of Manuscript (without track changes mode): Line 651-665, p27** |
| 4. | Costs of HPV vaccines. I am not well informed but I understand that one reason for the low HPV vaccination rates in Indonesia is the cost to the recipient and their families. Income levels are low at $350 US dollars per month. Western readers are accustomed to much higher incomes. This payment should be presented as per month each time it is included in the manuscript. | Thank you for pointing this out. We have added the durational specifier "per month" every time we mention the monthly income and funds to specify the financial status in Indonesia for the reasonable living costs of a family. We also added the ballpark costs for HPV vaccination in Indonesia along with analysis and comparison with other countries' prices, including Western countries. The relatively expensive vaccine cost in Indonesia is still a substantial and unresolved issue. However, some experts from our centers have proposed advocacy with pharmacies and the government to reduce the price and allow HPV vaccination to become a national universal vaccination program for adolescents at the primary school level.  **Current position of revision in new version of Manuscript (without track changes mode): Line 577-593, p24-25** |
| 5. | The manuscript is much too detailed. It is hard work reading the detail. I suggest much of the detailed data could be in Supplementary Tables. In addition much detailed data could be excluded from the narrative. | We agree with this suggestion, and it is a crucial point to make it clearer to read. We have moved the more detailed data to supplementary files. We also tried to exclude the detailed data from the narrative and visualize it as tables.  **Current position of revision in new version of Manuscript (without track changes mode): Line 266-385, p10-18** |
| 6. | I suggest that the authors include a comparison of the Indonesian guidelines for HPV vaccination with those of the United States Center for Disease Control. In the US HPV vaccination is recommended for young teenagers. The U.S. guide also recommend no HPV vaccination for people over the age of 26 (because of the likelihood of prior HPV infections). The situation may be very different in Indonesia. Sexual behaviour (the most common means of HPV transmission) is presumably different. This reviewer is not from the U.S., but many readers of this Journal are American. | We agreed. Thank you for pointing it out. We have added and emphasized the Indonesian guideline for HPV based on our Ministry of Health recommendation and compared it to several other countries such as the U.S., U.K., Australia, India, and Thailand.  **Current position of revision in new version of Manuscript (without track changes mode): Line 537-565, p23-24** |
| 7. | I suggest the authors include the risk of HPVs in male prostate cancer in their discussions section. This may alter the interest in HPV vaccination by male Indonesians. (see recent reviews in this Journal). Also women catch HPV infections from men. Based on this study, the HPV vaccinations are much lower in males than in females | We agree, and we thank the reviewer for their support on this matter.  We have added further emphasis on this issue in our discussions since this indicated that awareness of the male population about HPV and its related cancers is also crucial. The paradigm that HPV vaccination is not only for the female population but also for males is definitely new and underrepresented in Indonesia.  **Current position of revision in new version of Manuscript (without track changes mode): Line 610-636, p26** |

**#Reviewer Number 2**

We thank the reviewer for their constructive comments and suggestions, and we discuss these in the sequence below.

**Table 3.** Summary of revision based on Comments from Reviewer 2

| **No.** | **Comments** | **Response from the Author** |
| --- | --- | --- |
| 1. | **Abstract:**   a. The statement "No study explored Indonesian understanding of cervical cancer (CC) and the human 112 papillomaviruses (HPV) vaccine" --> It would be wiser if replaced with "Only few studies ...", knowing that there have been other studies conducted in Indonesia previously. b. It should be mentioned that the 400 respondents were recruited from cities in Jakarta Province, one of 34 provinces in Indonesia. Hence, it would be fair for the readers to consider the representativenes of the subjects compared to the whole urban communities of Indonesian population. | a Thank you so much for your prompt and detailed feedback. We have rephrased the phrase "no study" to "few studies" accompanied by a cited article. Although several studies have been conducted in Indonesia, our novelty is a knowledge gap between our study and prior research. One prior study in Indonesia more focused on parent's attitudes, beliefs, and uptake of the HPV vaccination [9], another only took samples from women in single primary care about knowledge and behavior regarding CC [10], a study was conducted only in junior high school students with a small sample pool [11], a study did research on women population about HPV vaccination and CC screening [12], Two studies only assessed knowledge, attitude, and acceptability of HPV vaccination among university students [13,14]. Therefore, our study is one of the few studies to address various subpopulations and interrelationships between knowledge, attitude, and practice about HPV, CC, and HPV vaccination in an Indonesian urban population.   **Current position of revision in new version of Manuscript (without track changes mode): Line 110, p5**   b We truly appreciate your thoughts. We have explicitly mentioned where our samples were recruited. We agree with your point that the representation of the subjects compared to the entire urban communities of the Indonesian population needs to be more clearly stated. Thus, we have explained Jakarta's status as Indonesia's capital city, and in the results section, we stated that our sample was recruited from Jakarta Province, one of 34 provinces in Indonesia and the largest metropolitan city in Indonesia.   **Current position of revision in new version of Manuscript (without track changes mode): Line 116, p5** |
| 2. | **Methods:**   a. Explain more detail about what form of questionnaire and platform used in the only survey b. Why the survey process took too long time for collecting 400 subjects? It is mentioned from March 2020 to August 2021. --> It needs to be explained because the time gap might influence the KAP due to enviromental changing in all aspect. c. Regarding the methods: what is the inclusion and exclusion criteria of the subjects? | a Thank you for your suggestion. Participants were recruited using the exponential non-discriminative snowball sampling technique. Questionnaires were made in Google Forms and distributed using virtual social media platforms such as Whatsapp, Line, Instagram, and people's networks. The questionnaire was filled out voluntarily by consent and anonymously. After filling out the questionnaire, we approached them through a message or called to make sure the subjects were eligible following our inclusion criteria, mainly to convince them they were Jakarta citizens.   **Current position of revision in new version of Manuscript (without track changes mode): Line 202-205, p8**   b Those timelines (March 2020 to August 2021) were for our whole research program. Indeed, a timeframe of 8-12 months is considered normal for a study analyzing the knowledge, attitude, and practice [15]. March 2020-December 2020 was the time to set our research proposal, ethical clearance, and other administrative trivial. In early 2021 we planned to conduct research exclusively for the student population (elementary school until senior high school level). However, the process was tricky due to schools locking down. Moreover, for students, we should explain and accompany them in filling out the questionnaire to ensure they understand. Therefore, we planned to open the questionnaire for all citizens of Jakarta. We admitted that the time gap might influence the KAP due to environmental changes, but we believe that the change is not significant since it is still during the pandemic. Moreover, no governmental policy changes nor new public health approaches are applied. During the pandemic, our government will not open the school lockdown for the school immunization month program, and thus, the opportunity to survey students would only open again in October 2021. However, private sectors like private vaccination centers or hospitals would still serve HPV vaccination. Thus, we thought the willingness to get vaccinated would not change during that period. Moreover, during the period, the minimum wages of our citizens did not change, especially in the city of Jakarta. If we view this topic from a cultural, educational, and social perspective, we do not see dynamic changes in those aspects in our city. Nevertheless, we agree that a more profound confirmation is needed to ensure that.   Other hindrances to the timeline are technical issues such as (1) abrupt interruptions to the sampling chain when one sample fails to provide a referral. The referral system purely relies on voluntary will, without any rewards or incentives for filling the questionnaire; (2) lack of cooperation and technical difficulties in contacting participants to check that the respondents do indeed live in Jakarta, following our inclusion criteria.   c Thank you for the kind feedback. We provide the inclusion and exclusion criteria we used in this study. Our sample has no stringent criteria because our target is the general population. However, all participants were selected by:   *Inclusion Criteria:*   1. The general public in all age groups ≥9 years (since our government program for HPV vaccination starts at nine years old) has diverse settings and community backgrounds, both male and female in Jakarta province. 2. The eligibility criteria for participants were urban citizens who had resided in metropolitan Jakarta, Indonesia, for at least six months (whether only moving to Jakarta for education or work, or native Jakarta residents as evidenced from their identity cards) [16]. 3. Having a basic level of literacy 4. Having given written informed consent to participate in the study and to be contacted directly to reveal their residency status   *Exclusion Criteria:*  The respondent did not fill out the questionnaire completely.  **Current position of revision in new version of Manuscript (without track changes mode): Line 191-194, p7-8** |
| 3. | **Conclusion:**  It is concluded that the knowledge was poor. What was the cutting score apllied to categorized the knowledge level? | Thank you for your asking to make our study clearer. Based on several prior publications on this topic, we decided on a cut-off to measure the knowledge, attitude, and practice regarding HPV infection, CC, and HPV vaccination. Referring to a previous study [17], in the type of questions with two and three answer choices, negative answers get a score of 0 while confirming answers get a score of 2. As seen in supplementary file S1:   a For example, question: "K1. Do you know that cervical cancer is a disease caused by a viral infection?" If a respondent says "yes," thus we give him/her +2 points for each question, and if he/she answers "no," we give a score of 0. b For example, question "K4. Can men transmit HPV?" If a respondent says "yes," we give him/her +2 points for each question, and if he/she answers "no" or "do not know," we give a score of 0.   To questions with four answer choices (Likert scale), negative answers get a score of 0, confirming answers get a value of 1, and highly confirming answers get a value of 2. As seen in supplementary file S1:   a For example, question: "A2. In your opinion, are you susceptible to HPV infection?" If a respondent says "yes," we give him/her a score of +2 points for each question, an answer of "maybe," was given a score of +1 point, and an answer of "unsure/no" was given a score of 0. b For example, question: "A5. I am worried that I/my partner/people close to me may develop cervical cancer in the future" If a respondent responded that he/she "absolutely agree," we give him/her a score of +2 points for each question; an answer of "agree," was given a score of +1 point, and an answer of "disagree/absolutely disagree" was given the score of 0.   .  This questionnaire's response can generally be dichotomized as good/positive/favorable (with +1 or +2 points) or poor/negative/unfavorable (0 points) answers. The results score of response items will be divided by the maximum score in each aspect (aspect 1 and 2) and multiplied by 100%— resulting in a final score for each aspect (aspect 1 and 2) and overall.  The detail of formula for the knowledge portion of the questionnaire was:   a Total score for aspect 1 knowledge = (K1 + K2 + … + K9) / 18 x 100% b Total score for aspect 2 knowledge = (K10 + K11 + … K21) / 24 x 100% c Total score for overall knowledge (aspect 1 + aspect 2) = (K1 + K2 + ….. + K21)/ 42 x 100%   The detail of formula for the attitude portion of the questionnaire was:   a Total score for aspect 1 attitude = (A1 + A2 + … + A5) / 10 x 100% b Total score for aspect 2 attitude = (A6 + A7 + … A12) / 14 x 100% c Total score for overall attitude (aspect 1 + aspect 2) = (A1 + A2 + ... + A12)/ 24 x 100%   The detail of the formula for the practice portion of the questionnaire was:  a Total score for overall practice= (P1 + P2 + ….. + P7)/ 14 x 100%  The cut-off for good knowledge, attitude, and practice for the questionnaire is a total score of ≥60% based on prior studies based on Bloom's cut-off points and modified by clinical judgment from the researcher [18–20]. Other studies used a cut-off score of 50% for good KAP [7]. A study used 80-100% correct response as good, 60-79% as satisfactory, and a score of <60% as poor [18].  We hope this is clear and can be understood. |

**#Reviewer Number 3**

**General Comments:** In general, the manuscript is interesting and provides evidence for enhancing effort of cervical cancer prevention, but need major revision, as follows.

**Author's Answer:** We appreciate the time you have committed to providing detailed and constructive feedback. We outline below our responses to these comments and, where appropriate, indicate what has been changed in the manuscript as a result

**Table 4.** Summary of revision based on Comments from Reviewer 3

| **No.** | **Suggestions** | **Response from the Author** |
| --- | --- | --- |
| 1. | **Title:**  Title should in line with the study population which is Jakarta citizens, not all urban communities in Indonesia. We suggest you to revise it. | Thank you for your feedback.  We admitted that stating that this study might represent all Indonesian urban communities might be questionable since it was only conducted in Jakarta. However, Jakarta is a pilot project city of the CC program implementation. Furthermore, our sample remains valuable, representative, and significant in picturing the Indonesian urban citizens since Jakarta is a city of transmigrants that receive a number of its population from cities all over Indonesia; hence the characteristic might be diverse and representative [16]. Most migrant citizens resided in Jakarta for a long time because of working and educational reasons. Approximately 42.5% of the total population of Jakarta were lifetime incoming migrants, while 7.3% of its population were recent incoming migrants. Lifetime in-migration for Jakarta is the third-largest percentage of its total population in Indonesia. However, recent outmigration from Jakarta surpasses incoming migrants; therefore, the net migration was −2.9%. This indicates that while Jakarta is still an attractive place for internal migrants, the number of outmigrants increases as they move to neighbor provinces such as West Java, Banten, and Lampung (Southern part of Sumatra) [16].  We have addressed this flaw of the study in our limitations section, and we have mentioned in the method section that our sample was taken from Jakarta, highlighted as one of the largest metropolitan cities in Indonesia.  Nevertheless, is it OK to still use a single urban city? We want to bring insight and novel in this article by enhancing urban city in the title. We emphasized the urban population in the title to make it better sound than Jakarta, whom only a city in Indonesia, and not all readers will be interested in reading.  **Current position of revision in new version of Manuscript (without track changes mode): Line 1-2, p1** |
| 2. | **Abstract:**   a. Background: Revise the word "no study explored…". There are many studies about HPV and cervical cancer. It's better if you write there is limitation of study about it, but you should provide the studies in the background b. Aim: what is the aim of the study? Association of KAP towards HPV infection, CC, and HPV vaccine? It's different with the result that is association of socio-demographic characteristics with KAP in terms of HPV infection, CC, and HPV vaccination. Make it clear. c. Method: add who the respondent are (Jakarta), sampling technique, how to get the respondent and sample size, how to collect data and when you did d. Result: Make it simple as aim of the study, not all result. We suggest to delete median, just straight to say high or poor (KAP) e. Conclusion: make it more specific related to the aim of the study. | a Thank you for your constructive feedback. We agree that this statement was too vague. We have rephrased the phrase "no study" to "few studies" accompanied by a cited article. Although several studies have been conducted in Indonesia, our novelty is the knowledge gap between our study and prior research. One prior study in Indonesia more focused on parent's attitudes, beliefs, and uptake of the HPV vaccination [9], another only took samples from women in single primary care about knowledge and behavior regarding CC [10], a study was conducted only in junior high school students with a small sample pool[11], a study did research on women population about HPV vaccination and CC screening [12], Two studies only assessed knowledge, attitude, and acceptability of HPV vaccination among university students [13,14]. Therefore, our study is one of the few studies to address various subpopulations and interrelationships between knowledge, attitude, and practice about HPV, CC, and HPV vaccination in an Indonesian urban population.   **Current position of revision in new version of Manuscript (without track changes mode): Line 110, p5**   b We agree, and we thank the reviewer for asking on this matter. We explained several aims in this study besides the association of KAP towards HPV infection, CC, and HPV vaccine. We attempted to explore: 1. The level of knowledge, attitude, and practice of HPV infection, CC, and HPV vaccination among urban citizens in Indonesia. 2. The difference of KAP of HPV infection, CC, and HPV vaccination based on gender. 3. The correlation between the level of knowledge, attitude, and practice related to HPV infection, CC, and HPV vaccination among urban citizens in Indonesia. 4. The correlation between the level of knowledge, attitude, and practice of HPV infection, CC, and HPV vaccination among urban citizens in Indonesia. 5. Contributing factors related to socio-demographic characteristics to KAP of HPV infection, CC, and HPV vaccination, how knowledge aspect can contribute to attitude aspect as a predictor, and how knowledge and attitude aspect can contribute to practice.   **Current position of revision in new version of Manuscript (without track changes mode): Line 111-112, p5**   c Thank you. We agree that this is an essential issue on the method that needs to be more explicit in the text.   1. We have added in the method section that our respondents were Jakarta citizens. The eligibility criteria for participants were urban citizens from diverse backgrounds aged ≥ 9 years, male or female who had resided in metropolitan city Jakarta, Indonesia, for at least six months [16], having a basic level of literacy, and having given written informed consent to participate in the study.   2. We have added in the method section that our sampling technique was exponential nondiscriminatory snowball sampling. In this technique, the first subject is recruited, and then he/she provides multiple referrals. Each new referral then provides more data for referral until there are enough subjects for the sample.   3. We have added in the method section that our respondents (sample size) were 400 people, which is beyond the minimum number as dictated by the statistical formula   4. We have added in the method section that we collected our data via the Google Forms online forms platform.   5. We have added in the method section that the whole research process was from March 2020 until August 2021.   **Current position of revision in new version of Manuscript (without track changes mode): Line 114-118, p5**   d Thank you for pointing this out. We have aligned the result with the aim of the study. The unnecessary data on the table have been moved to the supplementary file, and too detailed explanations have been removed. We did not delete tables 2-4 because we hope it can provide detailed data if readers want to know more about our findings.   Related your suggestion, thus:   1. The median data was deleted 2. We simply state if KAP is high/poor instead of mentioning a numerical data point   **Current position of revision in new version of Manuscript (without track changes mode): Line 119-131, p5**   e Thank you for the crucial and constructive suggestion. We have made it more specifically related to the aim of the study. All statements in this conclusion align with our findings related to aims. Additionally, recommendations have been constructed regarding the highlighted findings.   **Current position of revision in new version of Manuscript (without track changes mode): Line 132-133, p5** |
| 3. | **Background:**  Explain why you choose urban communities. Cervical cancer is a disease not only in urban, but also in rural communities. Provide evidence, such as comparison incidence or prevalence in urban and rural areas and related studies of KAP and cervical cancer. Again, word "no study explored…" should be revised | a Thank you for pointing this out. In this study, we choose urban citizens as a target because:   1. In Indonesia, CC screening and prevention has only been a national program since April 21, 2015 [21]. However, the vaccination program is still only centralized in urban areas. In 2016 vaccination programs were started city-wide in Jakarta, in 2017 in Yogyakarta and Surabaya, and in 2018 in Manado and Makassar [22].   2. As a pioneer of the HPV vaccination program, we are interested in measuring the KAP of HPV infection, CC, and HPV vaccination among urban citizens in Indonesia, particularly Jakarta. We start sampling in Jakarta as a foundation further to examine the readiness of these programs in rural areas. This is a new area for research in Indonesia (comparing the rural and urban particularly related prevalence of HPV infection, their KAP, and strategy-related programs).   3. Comparing the urban and rural incidence of CC, a study in the U.S. observed a higher prevalence of CC in urban counties (84.5% vs. 15.5%) compared to rural counties [23]. Another study in China reported that women from the urban population had a higher HPV prevalence (16.3%) than that of the rural population (13%) [24]. However, other studies also showed a higher prevalence in rural [11% (95% CI 4%-18%)] than urban citizens [10% (95% CI 8%-12%)] due to the disparity in vaccination and screening rate among rural residents [25]. No study has compared the situation in Indonesia yet. The only published data in 2019 related to this topic showed that the prevalence of HPV infection among urban women in Indonesia was 5.2% [26].   4. Urban and rural populations live in different socioeconomic environments, lifestyles, and living standards, which might explain the higher prevalence of overall HPV, oncogenic HPV, and multiple-genotype HPV infections in urban populations.   5. Thus, understanding HPV infection, CC, and HPV vaccine are essential for both of sex, notably in urban communities due to the complex situation related to a higher number of violence and crimes, smokers, drugs, economic and social status disparities, and sex workers [27,28]. A greater prevalence of alcoholism, smoking, drug consumption, and risky sexual behavior are risk factors for acquiring HPV infection [29–31].   6. Moreover, approximately 56.7% of Indonesia's population lived in urban areas in 2020 [32]   Thank you for pointing this out. Hence, in addressing your feedback, we have added evidence of comparison of incidence or prevalence of CC and HPV-related disease between urban and rural areas in the introduction section. However, we could not find published evidence about different KAP levels between urban and rural areas.  **Current position of revision in new version of Manuscript (without track changes mode): Line 144-152, p6**   b We agree that this is an important issue that needs to be more explicit in the text. The phrase "no study explored" has been revised into "few studies" and mentioned those scarcity studies previously conducted in Indonesia. We also have stated the gap of knowledge among them compared to our aims.   **Current position of revision in new version of Manuscript (without track changes mode): Line 169-180, p7** |
| 4. | **Methods:**   a. Describe how to spread the link/web in social media to invite the respondents. b. Explain how sample size is counted, there are several independent and dependent variables to be considered. c. Add information of time of data collection (is it more than a year from March 2020 – August 2021?). Is there any inclusion criteria/restriction of age? How a teenager of age of 11 years become respondent? d. In assessment tools, we suggest to move contributory from authors to specific section, not in method. e. In statistical analysis, add information of normality test, due to use of Spearman Correlation test. Add steps of multivariate logistic regression analysis, how the modeling constructed and how to get the final model (do you put the final model in the result?) | a We thank the reviewer for their support on this matter. In this research, we used exponential non-discriminative snowball sampling where the first subject is recruited and then he/she provides multiple referrals to her/his friend as an individual or in a group of social media. Each new referral then provides more data for referral until there are enough subjects for the sample. We used this method due to the COVID-19 pandemic, which is difficult to locate people and approach directly by face to face. We spread the questionnaire through Line, Whatsapp, and Instagram social media platforms   **Current position of revision in new version of Manuscript (without track changes mode): Line 202-205, p8**   b Thank you for addressing this issue on method. In order to make it clearer, in the method, we have added the detailed sample calculation along with the considered variable we used. To acquire prediction of minimum sample size, we used the sampling calculation based on a statistical basis using a formula of the differences in two proportion calculations in observational studies for descriptive and categorical data [3–6]. However, this paper did not detail the formula due to word number limitations following PLOS One guidelines. The detail of the formula for our calculation sample in this research was:   $n= \frac{{Z\alpha}^{2} \times P \times Q}{d^{2}}$   - n = the size of the sample used - α = generalization of wrong. A researcher decided the value (we used 0.05) - Zα = 1.96 (for α -value= 0.05). This is a standard value for alpha, and the value was obtained from the Z table normal curve - P = the proportion of categories or conditions or health states that become the point of interest (sought state). The value should be obtained from the literature, preliminary studies, or assumption. However, there is no previous research that was relevant to our research; the assumption of a proportion of 0.5 was used - Q = 1-P; thus, the value was 0.5 - d = the desired absolute level of accuracy or the precision of the estimate, namely the precision of the study as an error that is still acceptable (set by the researcher based on logical and enforceable principles), thus we decided the value was 0.05   $n= \frac{{1.96}^{2} \times0.5 \times0.5}{{0.05}^{2}}=196$  Nevertheless, 400 respondents participated in this study  **Current position of revision in new version of Manuscript (without track changes mode): Line 194-199, p8**  These are the considered variables in our study, and we have put this in our manuscript:  **Independent variables:**  The independent variables were socio-demographic information (age, education, occupations, salary per month, parent's education level, and religion) and several components of knowledge and attitude towards HPV infection, CC, and HPV vaccination.  **Dependent variables:**  The dependent variables were Knowledge, Attitude, and Practice levels towards HPV infection, CC, and HPV vaccination. However, these dependent variables can play as an independent variable when compared to each other as a predictor, for example, knowledge (independent variables as predictor) vs. attitude/ practice (dependent variable as outcome).  **Confounding variables:**  Possible confounding factors which can not be controlled through multivariate logistic regression analysis and still contribute to the level of KAP in this study were:   1. Government policy regarding HPV vaccination 2. Internet access to health information 3. Pandemic condition 4. Healthcare access facility 5. Occupation in more detail 6. Cultural, myth, beliefs, and religion may have an impact and influence, but they are not further assessed in this study 7. Caregiving patterns, childbearing, and habit in the family regarding health perception which not further assessed in this research 8. Social desirability bias [33]   **Current position of revision in new version of Manuscript (without track changes mode): Line 236-245, p9**   c Thank you. This is a good point.   1. Those timelines (March 2020 to August 2021) were for our whole research program. Indeed, an 8-12 months timeframe is considered normal for a study analyzing knowledge, attitude, and practices [15]. March 2020-December 2020 was the time to set our research proposal, ethical clearance, and other administrative trivia. In early 2021 we planned to conduct research exclusively for the student population (elementary school until senior high school level). However, the process was tricky due to schools locking down. Moreover, for students, we should explain and accompany them in filling out the questionnaire to ensure they understand. Therefore, we planned to open the questionnaire for all citizens of Jakarta. We admitted that the time gap might influence the KAP due to environmental changes, but we believe that the change is not significant since it is still during the pandemic. Moreover, no governmental policy changes nor new public health approaches are applied. During the pandemic, our government will not open the school lockdown for the school immunization month program, and thus, the opportunity to survey students would only open again in October 2021. However, private sectors like private vaccination centers or hospitals would still serve HPV vaccination. Thus, we thought the willingness to get vaccinated would not change during that period. Moreover, during the period, the minimum wages of our citizens did not change, especially in the city of Jakarta. If we view this topic from a cultural, educational, and social perspective, we do not see dynamic changes in those aspects in our city. Nevertheless, we agree that a more profound confirmation is needed to ensure that.   Other hindrances to the timeline are technical issues such as (1) abrupt interruptions to the sampling chain when one sample fails to provide a referral. The referral system purely relies on voluntary will, without any rewards or incentives for filling the questionnaire; (2) lack of cooperation and technical difficulties in contacting participants to check that the respondents do indeed live in Jakarta, following our inclusion criteria.   2. Our sample has no stringent criteria because our target is the general population. However, all participants were selected by:   *Inclusion Criteria:*   - The general public in all age groups ≥9 years (since our government program for HPV vaccination starts from age nine) with diverse settings and community backgrounds, both male and female in Jakarta province. - The eligibility criteria for participants were urban citizens who had resided in metropolitan city Jakarta, Indonesia, for at least six months (whether only domiciled to migrate to Jakarta only for education or work, or native Jakarta residents as evidenced from their identity cards) [16]. - Having a basic level of literacy - Having given written informed consent to participate in the study and to be contacted directly to find out their residency status   *Exclusion Criteria:*  The respondent did not fill out the questionnaire completely.  **Current position of revision in new version of Manuscript (without track changes mode): Line 191-194, p7-8**   3. Regarding the age group of 11-20 yo, we made a range group to stratify the age group starting from 11 years. However, in our inclusion criteria, everyone over the age of 9 can also be a respondent since they are a target group for HPV vaccination based on our government program and recommendation. Moreover, this teenager may fill out the questionnaire under guidance from parents and correspondence with us as a researcher. However, in our study, there is no teenager of 11 years old or younger included in this study; the youngest participant was 13 years old.  d This is a good point. We agree to move the questionnaire construction contributors to a specific author contribution section.   **Current position of revision in new version of Manuscript (without track changes mode): Line 79-82, p3**   e Thank you for pointing this out. We have added a section on the normality test using a Kolmogorov-Smirnov test, which showed that our data was skewed (abnormal in distribution); therefore, we used the Spearman correlation test. Kolmogorov-Smirnov for aspects 1 and 2 of knowledge, overall knowledge, aspects 1 and 2 of attitude, overall attitude, and overall practice score was 0.000, indicating substantially skewed distribution.   Regarding the multivariate logistic regression analysis, we will therefore explain how we conducted this statistical analysis and how the modeling was constructed until the final model. At first, we have put the final model on the result as tables 6-9 (previous version) and tables 3-6 (revised version); please see the variables which have significant adjusted OR. We did not remove the insignificant predictors to see the insignificant and significant contributors from the bivariate until the multivariate regression logistic model was constructed. The steps were:   1. We consider the point of interest that is related to risk factors/ predictors related to aspects of socio-demographical characteristics to contribute in terms of: 1) Good knowledge and other related aspects of knowledge (understanding that CC can be prevented by vaccination and being aware of HPV vaccine availability in Indonesia) 2) Positive attitudes and other related aspects of attitudes (worrying about a partner or closed family will get CC and thinking that the partner or closed family must be vaccinated against HPV) 3) Favorable/appropriate practices and other related aspects of practices (have been vaccinated against HPV at least one dose and intention and readiness to take HPV vaccination) 2. We also consider the point of interest related to risk factors/predictors related to proper aspects of knowledge, attitude, and practice to contribute in terms of willingness and readiness to get the HPV vaccination. 3. We performed bivariate analysis using Chi-Square or Fischer's exact test and Mantel-Haenszel odds ratio estimate tests to calculate the unadjusted OR. 4. Any associated factors with p≤0.20 were deemed eligible for inclusion in the multivariate analysis model [34–37]. Some authors used cut-off p≤0.25 as cut-off eligibility for multivariate analysis [38–42], but we chose p≤0.20 5. A multivariate logistic regression analysis was constructed in SPSS using the backward method with the steps:   analyze 🡪 regression 🡪 binary logistic 🡪 putting interesting variables (point of interest) to the dependent box 🡪 putting predictors/contributing factors as covariates 🡪 choosing "Backward: L.R. method" 🡪 choosing "categorical covariates" 🡪 change reference category as a first 🡪 continue 🡪 choose and run the options in Hosmer and Lemeshow for "goodness-of-fit test" and choose "CI for EXP(B)" 🡪 continue 🡪 save 🡪 probabilities on predicted values box 🡪 OK 🡪 running the process   6. Interpretation of results    1) In the table of "Variables in Equation," we see the significance of columns for each step until the final steps of logistic regression using backward: L.R. methods to know p-value for Wald test for each variable's coefficient.    2) Seeing the Exp(B) and 95%CI lower-upper columns to know OR    3) In the table of "Hosmer and Lemeshow Test," we see the result of the Hosmer and Lemeshow test to measure the quality of regression. The null hypothesis of the Hosmer and Lemeshow test is that there is no difference between the observed value and the expected value. We found that all p-values in this test were >0.05 so that the null hypothesis was accepted, which means that there is no difference between the observed and expected values. Thus the obtained equation is well calibrated.   **Current position of revision in new version of Manuscript (without track changes mode): Line 308-323, p13-14** |
| 5. | **Result:**   a. Table 2 – 4 is not needed, its only step to get table 4. We suggest deleting table 2-4. b. Table 6-9: are they the final model? You should give explanation. Put only the significant variables in the final model. make table 9 simpler. | a We appreciate the time you have committed to providing detailed and constructive feedback. We agree with this suggestion, but we still consider including this data results by moving it to supplementary data because it is still beneficial and valuable for readers to comprehend the full results of our survey regarding knowledge, attitude, and practice between males and females.   **Current position of revision in new version of Manuscript (without track changes mode): Line 281-291, p12**   b Thank you for asking about this issue. Yes, our multivariate logistic analysis in Table 6-9 (in the previous version of the manuscript) is the final prediction model. Univariate analyses of potential factors associated with the dependent variables of KAP were done. Any factors related to each outcome with p≤0.20 were considered for inclusion as fixed effects in the step-wise multiple regression analyses model [34]. The final model was developed by performing the backward L.R. method in the variables selection process [38]. In the final model, a p-value of <0.05 indicated statistical significance.   In the previous version of the manuscript, we have put only significant clinical variables with p<0.05 in the final model (please see the "adjusted OR (95%CI)" column). We wrote the OR with "-" and p-value "n/a" for insignificant variables. However, we still possessed all considered variables in table 6-9, specifically in the column "unadjusted OR (95%CI)", since to the best of our knowledge, in performing a logistic regression model, we need to start with univariate analysis and presenting which variables are insignificant (p>0.20) and which ones are with a p-value <0.20 to be included in step-wise multiple regression analyses model [34].  Regarding how we present the final model in table 6-9, in our perspective, it has been made simpler by still considering all aspects examined. We followed several prior studies that use similar data presentations such as:   1. **Title:** Young adults' human papillomavirus related knowledge: source of medical information matters [43]  2. **Title:** Knowledge, attitudes, and practices toward cervical cancer prevention among women in Kampong Speu Province, Cambodia [44]      3. **Title:** Knowledge and Attitude toward Human Papillomavirus Infection and Vaccination among Thai Women: A Nationwide Social Media Survey [45]     **Current position of revision in new version of Manuscript (without track changes mode): Line 335-385, p14-18** |
| 6. | **Discussion:**   a. The age of respondents majority in age of 11-30 (80%), the must a specific recommendation in this age b. Add limitation of the study in terms of sampling technique (snowball), that can not fully represent the population of Jakarta citizens/urban communities. The limitation of characteristic especially age, so result of the study may not be generalized to all ages. | a Thank you for taking the time to provide some feedback for our manuscript. We have added recommendations in the conclusion section, which specifies that this is most important for Indonesians aged 11-30 years—the most critical advice for this age group is to get quadrivalent or nonavalent HPV vaccination; (or bivalent vaccines, if other options are absent). People with age <15 years can still use two-dose vaccination and above it should be vaccinated with three doses (treated as catch-up vaccination). Susceptible people ≥15 years, regardless of their sexual activity and infection status, may still do catch-up vaccination with three doses of HPV vaccine due to the benefit of the vaccine to prevent infection with uninfected HPV serotypes. This age group is highly recommended to get HPV DNA testing to get an early diagnosis. Alternatively, women in this age group can also do the IVA test with or without following a pap smear to screen CC status.   **Current position of revision in new version of Manuscript (without track changes mode): Line 681-688, p28**   b Thank you for your kind suggestion. We have limitations in terms of sampling technique and online survey, which can not fully represent Jakarta and Indonesian urban citizens. We have also mentioned age as one limitation which can not be generalized to all ages.   **Current position of revision in new version of Manuscript (without track changes mode): Line 662-663, p27** |
| 7. | **Conclusion:**  Elaborate the most interesting findings related to the aim of the study. We suggest not to use term "domain" that is not easy to understand. | a We agree, and we thank the reviewer for their support on this matter. The most interesting findings would be that: 1. Urban citizens become the center of our interest. Few studies focused on the urban community in terms general public. Prior studies mainly researched women, student, and healthcare professional populations. They did not assess the location of the sample. Our study can be a pioneer source to assess another community in the future, such as comparing rural and urban residents. 2. We pictured the socio-demographical background of our participants comprehensively, completely, and holistically by showing age groups, young/age group, formal education, level of education, occupations, employment, salary per month, formal education of fathers and mothers, level of education of fathers and mothers, and religion. 3. We established the association between knowledge, attitude, and practice about HPV infection, CC, and HPV vaccination. 4. We divided the component of knowledge and attitude into two aspects: related to HPV infection and CC and HPV vaccination. It is interesting to understand more for understanding HPV-related disease issues. 5. Socio-demographic profile and its influence on knowledge, attitude, and practice about HPV infection, CC, and HPV vaccination were presented. 6. The odds ratio for predicting good knowledge, attitude, and practice becomes a unique value to understand significant contributing factors to improve the program. 7. The curve of linear regression to know the association between knowledge, attitude, and practice along with inter aspects were captured eye-catching   **Current position of revision in new version of Manuscript (without track changes mode): Line 673-696, p28-29**   b Yes, we agree that this word might be too vague. We considered to changed the "domain" term into "aspect." |

That is all the changes we have made. Because there are many revisions in our manuscript, which resulted in quite a lot of changes in the reference citation arrangement and improvement in grammar, hence, in preventing missed data, we ask the reviewer and editor to use this latest version of our Word document for the following publication process. We also have proofread for any grammatical and wording issues in the revision process. Please use the Track Changes feature inherent to your word processing software to view revised sentences and phrases.

We hope that this revised manuscript will meet your requirements and be granted the opportunity to be published in PLOS ONE.

Thank you

**Sincerely,**

**Hariyono Winarto, MD, Gynaecologist-Oncologist, PhD**

Division of Gynecology Oncology, Department of Obstetrics and Gynecology, Faculty of Medicine, Universitas Indonesia and Dr. Cipto Mangunkusumo National Referral Hospital, Jakarta, Indonesia

**References:**

1.	Raina SK. Establishing association. Indian J Med Res [Internet]. 2015 Jan;141(1):127. Available from: https://pubmed.ncbi.nlm.nih.gov/25857507

2.	Kirchherr J, Charles K. Enhancing the sample diversity of snowball samples: Recommendations from a research project on anti-dam movements in Southeast Asia. PLoS One. 2018;13(8):1–17.

3.	Pourhoseingholi MA, Vahedi M, Rahimzadeh M. Sample size calculation in medical studies. Gastroenterol Hepatol from Bed to Bench. 2013;6(1):14–7.

4.	Charan J, Biswas T. How to calculate sample size for different study designs in medical research? Indian J Psychol Med. 2013;35(2):121–6.

5.	Dahlan MS. Besar Sampel dalam Penelitian Kedokteran dan Kesehatan [In Indonesian]. 5th ed. Jakarta: Epidemiologi Indonesia; 2019. 1–287 p.

6.	Yitagesu H, Samuel Y, Tariku L. Knowledge, attitude and practice for cervical cancer prevention and control among women of childbearing age in Hossana Town, Hadiya zone, Southern Ethiopia: Community-based cross-sectional study. PLoS One. 2017;12(7):181–200.

7.	Almazrou S, Saddik B, Jradi H. Knowledge, attitudes, and practices of Saudi physicians regarding cervical cancer and the human papilloma virus vaccine. J Infect Public Health [Internet]. 2020;13(4):584–90. Available from: https://doi.org/10.1016/j.jiph.2019.09.002

8.	Baskar K, Lakshmi T. Knowledge, Attitude and Practices Regarding HPV Vaccination among Undergraduate and Postgraduate Dental Students in Chennai. J Pharm Res Int. 2020;32(17):95–100.

9.	Wijayanti KE, Schütze H, MacPhail C. Parents' attitudes, beliefs and uptake of the school-based human papillomavirus (HPV) vaccination program in Jakarta, Indonesia – A quantitative study. Prev Med Reports [Internet]. 2021;24:101651. Available from: https://doi.org/10.1016/j.pmedr.2021.101651

10.	Winarto H, Mitzy B. S, Widodo AB, Kurniawan A, Phallaphi YR. Cervical Cancer Related Knowledge, Attitude and Behaviour Among Women in Makasar District Primary Health Care Centre in 2018. Open Public Health J. 2019;12(1):337–41.

11.	Lismidiati W, Emilia O, Widyawati W. Human Papillomavirus (HPV) Health Savings as an Alternative Solution: HPV Vaccination Behavior in Adolescents. Asian Pacific J Cancer Prev. 2021;22(2):471–6.

12.	Endarti D, Satibi, Kristina SA, Farida MA, Rahmawanti Y, Andriani T. Knowledge, perception, and acceptance of HPV vaccination and screening for cervical cancer among women in Yogyakarta Province, Indonesia. Asian Pacific J Cancer Prev. 2018;19(4):1105–11.

13.	Khatiwada M, Kartasasmita C, Mediani HS, Delprat C, Van Hal G, Dochez C. Knowledge, Attitude and Acceptability of the Human Papilloma Virus Vaccine and Vaccination Among University Students in Indonesia. Front Public Heal. 2021;9(June):1–9.

14.	Wibisono P, Widjaja AG, Vatvani AD, Velies DS. Knowledge, attitude and practice of HPV vaccination in undergraduate medical and nursing students. Ann Oncol Abstr Gynaecol Cancers. 2017;28(November):x90.

15.	Goutille F, Crini V, Jullien P. Knowledge, Attitudes and Practices for Risk Education: how to implement KAP surveys - Guideline for KAP survey managers [Internet]. 2009. 84 p. Available from: http://www.hiproweb.org/uploads/tx_hidrtdocs/GuideCAPGB.pdf%5Cnhttp://www.handicap-international.org.uk/Resources/Handicap International/PDF Documents/HI Associations/KAPRiskEducation_2009.pdf

16.	Martinez R, Masron IN. Jakarta: A city of cities. Cities. 2020;106:102868.

17.	Di Giuseppe G, Abbate R, Liguori G, Albano L, Angelillo IF. Human papillomavirus and vaccination: Knowledge, attitudes, and behavioural intention in adolescents and young women in Italy. Br J Cancer. 2008;99(2):225–9.

18.	Ali AN, Kar Mun L, Prajapati SK, Zahid Iqbal M, Zulfikar Ahmed N. Cervical cancer, its screening and vaccination: a KAP study among female healthcare students in a Private University, Malaysia. MOJ Bioequivalence Bioavailab. 2018;5(5):275–82.

19.	Adejuyigbe FF, Balogun BR, Sekoni AO, Adegbola AA. Cervical cancer and human papilloma virus knowledge and acceptance of vaccination among medical students in Southwest Nigeria. Afr J Reprod Health. 2015;19(1):140–8.

20.	Kaliyaperumal KI. Guideline for Conducting a Knowledge, Attitude and Practice (KAP) Study. AECS Illum. 2015;7–9.

21.	Kementerian Kesehatan Republik Indonesia. Program Nasional Gerakan Pencegahan dan Deteksi Dini Kanker Leher Rahim dan Kanker Payudara [In Indonesian] [Internet]. Jakarta: Kementerian Kesehatan Republik Indonesia; 2015. 1–47 p. Available from: http://p2ptm.kemkes.go.id/uploads/2016/10/Panduan-Program-Nasional-Gerakan-Pencegahan-dan-Deteksi-Dini-Kanker-Kanker-Leher-Rahim-dan-Kanker-Payudara-21-April-2015.pdf

22.	Kementerian Kesehatan Republik Indonesia. Keputusan Menteri Kesehatan Republik Indonesia Tentang Pelaksanaan Demonstrasi Pemberian Imunisasi Human Papillomavirus di Kota Surabaya, Kota Manado, dan Kota Makassar [In Indonesian] [Internet]. Kementerian Kesehatan Republik Indonesia, HK.01.07/MENKES/194/2017 Jakarta, Indonesia: Kementerian Kesehatan Republik Indonesia; 2017 p. 5. Available from: http://hukor.kemkes.go.id/uploads/produk_hukum/KMK_No._HK_.01_.07-MENKES-194-2017_ttg_Demonstrasi_Pemberian_Imunisasi_HPV_.pdf

23.	Yu L, Sabatino SA, White MC. Rural – Urban and Racial / Ethnic Disparities in Invasive Cervical Cancer Incidence in the. Prev Chronic Dis [Internet]. 2019;16:180447. Available from: http://dx.doi.org/10.5888/pcd16.180447

24.	Baloch Z, Yuan T, Yindi S, Feng Y, Tai W, Liu Y, et al. Prevalence of genital human papillomavirus among rural and urban populations in southern Yunnan province, China. Brazilian J Med Biol Res = Rev Bras Pesqui medicas e Biol. 2016;49(6):e5254.

25.	Lee M, Gerend MA, Adjei Boakye E. Rural–Urban Differences in Human Papillomavirus Vaccination Among Young Adults in 8 U.S. States. Am J Prev Med [Internet]. 2021;60(2):298–9. Available from: https://doi.org/10.1016/j.amepre.2020.07.023

26.	Handayani S, Dewi RM, Wibowo HA, Dany F, Kipuw NL, Adelina R, et al. Cervical Cancer Risk Factor: HPV Infection Among Indonesian Urban Women. 2020;22(Ishr 2019):452–5.

27.	Skinner SR, Marino J, Rosenthal SL, Cannon J, Doherty DA, Hickey M. Prospective cohort study of childhood behaviour problems and adolescent sexual risk-taking: gender matters. Sex Health. 2017;14(6):492–501.

28.	Wallace SA, Neilands TB, Sanders Phillips K. Neighborhood context, psychological outlook, and risk behaviors among urban African American youth. Cultur Divers Ethnic Minor Psychol. 2017;23(1):59–69.

29.	Pedrana AE, Pina J, Padmawati RS, Zuhrina R, Lazuardi L, Lim MSC, et al. A quasi-experimental text messaging trial to improve adolescent sexual and reproductive health and smoking knowledge in Indonesia. Sex Health. 2020;17(2):167–77.

30.	Bonar EE, Walton MA, Epstein-Ngo QM, Zimmerman MA, Blow FC, Cunningham RM. Two-Year Trajectories of Sexual Risk Behaviors Among Drug-Using Adolescents and Emerging Adults in an Urban Community. AIDS Behav. 2017;21(7):2069–78.

31.	Amoo EO, Adekeye OA, Omumu F, Akinpelu OO, Ajayi MP, Olawande T, et al. Drug use and high-risk sexual behavior among school-going adolescents in Nigeria. Open Access Maced J Med Sci. 2020;8:256–61.

32.	BPS-Statistics Indonesia. Statistical Yearbook of Indonesia [in Indonesian] [Internet]. Directorate of Statistical Dissemination, editor. Badan Pusat Statistik/BPS-Statistics Indonesia. Jakarta: Badan Pusat Statistik/BPS-Statistics Indonesia; 2021. 1–804 p. Available from: https://www.bps.go.id/publication/2020/04/29/e9011b3155d45d70823c141f/statistik-indonesia-2020.html

33.	Mondal H, Mondal S. Social desirability bias: A confounding factor to consider in survey by self-administered questionnaire. Indian J Pharmacol [Internet]. 2018;50(3):143–4. Available from: https://pubmed.ncbi.nlm.nih.gov/30166752

34.	Juvanhol LL, Lana RM, Cabrelli R, Bastos LS, Nobre AA, Rotenberg L, et al. Factors associated with overweight: Are the conclusions influenced by choice of the regression method? BMC Public Health. 2016;16(1):1–9.

35.	Fenlon C, O'Grady L, Doherty ML, Dunnion J, Shalloo L, Butler ST. The creation and evaluation of a model predicting the probability of conception in seasonal-calving, pasture-based dairy cows. J Dairy Sci [Internet]. 2017;100(7):5550–63. Available from: http://dx.doi.org/10.3168/jds.2016-11830

36.	Adli I, Widyahening IS, Lazarus G, Phowira J, Azzahra L, Ariffandi B, et al. Knowledge, attitude, and practice related to the COVID-19 pandemic among undergraduate medical students in Indonesia: a nationwide cross-sectional study. medRxiv [Internet]. 2021;2021.07.05.21260021. Available from: https://www.medrxiv.org/content/10.1101/2021.07.05.21260021v1%0Ahttps://www.medrxiv.org/content/10.1101/2021.07.05.21260021v1.abstract

37.	Boltes S, Monteiro-soares M, Henriques T, Monteiro E, Isabel C, Pinto F. Derivation of a clinical decision rule for predictive factors for the development of pharyngocutaneous. Braz J Otorhinolaryngol [Internet]. 2015;81(4):394–401. Available from: http://dx.doi.org/10.1016/j.bjorl.2014.09.009

38.	Dahlan MS. Analisis Multivariat Regresi Logistik [In Indonesian]. 2nd ed. Jakarta: Epidemiologi Indonesia; 2019. 1–175 p.

39.	Bursac Z, Gauss CH, Williams DK, Hosmer DW. Purposeful selection of variables in logistic regression. Source Code Biol Med [Internet]. 2008 December 16;3:17. Available from: https://pubmed.ncbi.nlm.nih.gov/19087314

40.	Zhu Y, Fan L, Zhang H, Wang M, Mei X, Hou J. Is the Best Evidence Good Enough : Quality Assessment and Factor Analysis of Meta- Analyses on Depression. 2016;1–15.

41.	Mascarel ADE, Broutet N, Marais A, Samoyeau R, Salamon R, Me F, et al. cagA Status and Eradication Treatment Outcome of Anti- Helicobacter pylori Triple Therapies in Patients with Nonulcer Dyspepsia. 2001;39(4):1319–22.

42.	Arba A. Knowledge of Essential Newborn Care and Associated Factors among Nurses and Midwives : A Cross-Sectional Study at Public Health Facilities in Wolaita Zone , Southern Ethiopia , 2019. 2020;2020.

43.	Benavidez G, Asare M, Lanning B, Ylitalo K, Fakhoury C, Thompson N, et al. Young adults' human papillomavirus–related knowledge: source of medical information matters. Public Health. 2020;182:125–30.

44.	Touch S, Oh JK. Knowledge, attitudes, and practices toward cervical cancer prevention among women in Kampong Speu Province, Cambodia. BMC Cancer. 2018;18(1):1–8.

45.	Likitdee N, Kietpeerakool C, Chumworathayi B, Temtanakitpaisan A, Aue-Aungkul A, Nhokaew W, et al. Knowledge and Attitude toward Human Papillomavirus Infection and Vaccination among Thai Women: A Nationwide Social Media Survey. Asian Pacific J Cancer Prev. 2020;21(10):2895–902.
